# Stromal and Immune Cell Dynamics in Tumor Associated Tertiary Lymphoid Structures and Anti-Tumor Immune Responses

**DOI:** 10.3389/fcell.2022.933113

**Published:** 2022-07-08

**Authors:** Alessandra Rossi, Beatrice Belmonte, Silvia Carnevale, Antonietta Liotti, Veronica De Rosa, Sebastien Jaillon, Silvia Piconese, Claudio Tripodo

**Affiliations:** ^1^ Department of Internal Clinical Sciences, Anesthesiology and Cardiovascular Sciences, Sapienza University of Rome, Rome, Italy; ^2^ Tumor Immunology Unit, Department of Sciences for Health Promotion and Mother-Child Care “G. D’Alessandro”, University of Palermo, Palermo, Italy; ^3^ RCCS Humanitas Research Hospital, Milan, Italy; ^4^ Istituto per l’Endocrinologia e l’Oncologia Sperimentale, Consiglio Nazionale Delle Ricerche, Naples, Italy; ^5^ Department of Biomedical Sciences, Humanitas University, Milan, Italy; ^6^ IRCCS Fondazione Santa Lucia, Unità di Neuroimmunologia, Rome, Italy; ^7^ Laboratory Affiliated to Istituto Pasteur Italia—Fondazione Cenci Bolognetti, Rome, Italy; ^8^ Histopathology Unit, FIRC Institute of Molecular Oncology (IFOM), Milan, Italy

**Keywords:** TLS, Treg, Tfh, neutrophils, tumor stroma

## Abstract

Tertiary lymphoid structures (TLS) are ectopic lymphoid organs that have been observed in chronic inflammatory conditions including cancer, where they are thought to exert a positive effect on prognosis. Both immune and non-immune cells participate in the genesis of TLS by establishing complex cross-talks requiring both soluble factors and cell-to-cell contact. Several immune cell types, including T follicular helper cells (Tfh), regulatory T cells (Tregs), and myeloid cells, may accumulate in TLS, possibly promoting or inhibiting their development. In this manuscript, we propose to review the available evidence regarding specific aspects of the TLS formation in solid cancers, including 1) the role of stromal cell composition and architecture in the recruitment of specific immune subpopulations and the formation of immune cell aggregates; 2) the contribution of the myeloid compartment (macrophages and neutrophils) to the development of antibody responses and the TLS formation; 3) the immunological and metabolic mechanisms dictating recruitment, expansion and plasticity of Tregs into T follicular regulatory cells, which are potentially sensitive to immunotherapeutic strategies directed to costimulatory receptors or checkpoint molecules.

## Introduction

Human solid cancers have been traditionally classified into subtypes based on disease stage and histology for prognostic purposes and selection of clinical treatments. In the past decades the development of new therapeutic strategies and the accumulating knowledge on tumor biology has called for a more comprehensive classification system that takes into account the complexity of the disease in terms of qualitative heterogeneity and response to therapies. Indeed, it is now well recognized that tumors are heterogeneous entities where the stromal components (fibroblasts, blood vessels and infiltrating leukocytes) are major determinants of disease progression and can be therapeutic targets along with transformed cells. In this view, a detailed characterization of the tumor microenvironment as a whole represents a pivotal tool to fully exploit the potential of new therapies and ultimately improve patients’ survival and quality of life.

Infiltrating leukocytes often outnumber transformed cells in tumor masses and can grow in organized structures resembling secondary lymphoid organs (SLO), known as tumor associated tertiary lymphoid structures (TLS). The number and qualitative composition of TLS can vary significantly among tumor types but there is now consensus about the overall positive effects they exert on prognosis and response to immunotherapy. It is therefore important to include the immune profile when characterizing tumors in order to make appropriate decisions in terms of therapy and disease clinical management. In this review the general features of tumor-associated TLS and of their formation will be described. The role of the TLS-associated cell types, mainly T and B lymphocytes subsets and myeloid cells, will also be discussed and related to the induction of tumor specific immunity and to the prognostic significance of these structures.

## Tertiary Lymphoid Structures: General Features and Prognostic Significance in Cancer

TLS are ectopic lymphoid organs that originate within nonlymphoid tissues during chronic inflammatory conditions, such as persistent pathogen infection, autoimmune disorders, allograft rejection and cancer (Ansel and Cyster, 2001; Aloisi and Pujol-Borrell, 2006). TLS, also named tertiary lymphoid organs (TLO), develop in peripheral tissues in close proximity to pathological loci, under stress conditions and as the result of persistent antigen stimulation (Sautes-Fridman et al., 2016). TLS structure resembles that of conventional secondary lymphoid organs (for example, lymph nodes) including B cell zones, T cell zones, marginal zones with activated macrophages and dendritic cells, reticular fibroblast cell networks and vasculature permissive to immune cell extravasation (Aoyama et al., 2021). However, while lymph nodes are encapsulated, TLS represent a congregation of immune and stromal cells confined within an organ or tissue (Aoyama et al., 2021). TLS form *de novo* in the microenvironment of solid tissues in response to prolonged inflammatory stimuli including cancer and may dissipate upon the resolution of inflammation (Moyron-Quiroz et al., 2004). TLS can additionally foster tumor antigen presentation, T cell activation and clonal expansion (Joshi et al., 2015; Zhu et al., 2015), activation of antigen-presenting cells (APC) (Hughes et al., 2016), germinal center (GC) formation (Silina et al., 2018), and B cell class switching (Schroder et al., 1996). Various types of innate and adaptive immune cells comprising macrophages, DCs, mast cells, as well as T and B cells, are recruited and activated by inflammatory mediators, especially cytokines, chemokines and adhesion molecules to the site of persistent inflammation and participate to the formation of TLS (Weinstein and Storkus, 2015; Helmink et al., 2020). As compared to secondary lymphoid structures, TLS exhibit a fair amount of heterogeneity in terms of cellular composition and topographical localization within tissue types.

TLS are lymphoid aggregates characterized by two distinct B and T compartments. Fibroblastic reticular cell networks, PNAd^+^ High Endothelial Venules (HEVs) and follicular dendritic cells are found within T cell zones. On the other hand, B compartment consists of B cell follicles primarily composed of naïve B cells with evidence for class switching and reactive GC in B cell zones, and expression of the enzyme activation-induced cytidine deaminase (AID), required for the initiation of somatic hypermutation and immunoglobulin gene class switching (Muramatsu et al., 2000; Neyt et al., 2012; Dieu-Nosjean et al., 2014; Jones et al., 2016). At an early stage, TLS show a different composition and primarily consist of T and B cells without formation of follicles with evidence of GC.

Tumor-associated TLS from different tumor types vary in cellular composition and organization. In fact, they variably contain B lymphocytes with immature, naïve, activated, memory and plasma cell phenotypes (Sautes-Fridman et al., 2019). In non-small cell lung cancer (NSCLC), tumor-associated TLS include large numbers of mature dendritic cells DC-LAMP^+^, which are not detected in lung metastatic neoplasms. The density of mature DC within primary lung tumor TLS has been found to be related to the amount of intratumoral Th1 and CD8^+^ T cell infiltration, and positively influences the effectiveness of antitumoral cytotoxic immune response. Thus, these findings seem to indicate mature DC as possible biomarkers of clinical outcome and as predictors of long-term survival (Dieu-Nosjean et al., 2008; Dieu-Nosjean et al., 2014; Goc et al., 2014). TLS in breast cancer commonly contain T helper follicular (Tfh) cells (Gobert et al., 2009), whereas those associated with prostate and lung metastatic colon cancer contain large numbers of regulatory T cells (Tregs) (Kang et al., 2021). A high density of Tfh is commonly reported in TLS in breast cancer (Rubin and Kan, 1985), although it is also documented in those associated with prostatic adenocarcinoma (Garcia-Hernandez et al., 2017) and colorectal adenocarcinoma with pulmonary metastasis (Schweiger et al., 2016). Tfh and Treg cells have been shown to act in a complex balance in TLS formation and influence the composition of the neoplastic chronic inflammatory microenvironment. Gu-Trantien et al. demonstrated that, in breast cancer models, Treg accumulation occurs in response to antigen-induced IL-2 production and contributes to the suppression of Th1-mediated adaptive immune response. Later, the IL-2-depleted microenvironment induces the differentiation of some activated CD4^+^ T cells into CXCL13-producing Tfh, capable of sustaining TLS formation and tumor-infiltrating B cell recruitment (Gu-Trantien et al., 2013).

Well-organized TLS have been described in several solid tumors with different topographic locations either in close contact with malignant cells and in peritumoral areas, and are generally considered a favorable prognostic factor (Sautes-Fridman et al., 2016; Sautes-Fridman et al., 2019). Several studies documented the presence of intratumoral TLS with a non-classical organization (without the evidence of discrete T- and B-cell compartments) in hepatocarcinoma (Finkin et al., 2015) and renal cell carcinoma presenting pulmonary metastasis (Remark et al., 2013). Other studies highlighted peritumoral TLS in proximity to the tumor-invasive margin (Munoz-Erazo et al., 2020).

TLS are privileged sites where the activation and the maintenance of local and systemic T and B cell response against tumor antigens occur (Heinhuis et al., 2019) and decelerate neoplastic progression (Dieu-Nosjean et al., 2016). Several studies reported their presence in NSCLC (Germain et al., 2014), colorectal cancer (McMullen et al., 2010; Di Caro et al., 2014), breast (Martinet et al., 2011), pancreatic (Hiraoka et al., 2015; Castino et al., 2016) and gastric carcinomas (Hennequin et al., 2016), oral cancer (Wirsing et al., 2018) as well as ovarian cancer (Milne et al., 2009) and melanoma (Ladanyi et al., 2007; Engelhard et al., 2018; Sautes-Fridman et al., 2019).

Numerous preclinical and clinical studies attribute varied roles to TLS in accordance with their structure or with the neoplastic characteristics related to either the site of origin or the location of the neoplasm. Indeed TLS promote a vigorous immune response intratumorally, constituting a barrier to disease progression, and also modulate immunity in the tumor microenvironment (Engelhard et al., 2018). The presence of TLS correlates to the activation of the immune response and for this reason they are considered a biomarker for stratifying untreated cancer patients’ survival risk and an intriguing and promising target for predicting the efficacy of new immunotherapies (Colbeck et al., 2017a; Helmink et al., 2020). Although the prognostic impact of tumor-associated TLS has been extensively investigated, the immune response that occurs in TLS and the significance of their prognostic role remain incomplete. Several studies have indicated a significant correlation between TLS density and higher rates of disease-free survival and overall patient survival (Sautes-Fridman et al., 2019), although there are exceptions (Figenschau et al., 2015; Finkin et al., 2015). However, it is observed that patient survival depends strictly on TLS’ classical or non-classical organization and also peri- and intratumoral localization. Tumor-associated TLS and/or stromal cells (as high endothelial venules, HEVs) can be extratumoral, positioned at or outside the tumor invasive margin, or intratumoral, located within the true tumor mass or tumor nests. The importance of TLS/HEVs in enabling infiltration of T cells into the tumor has been demonstrated. Indeed, unlike intratumoral structures, extratumoral HEVs were not associated with increased tumor-infiltrating lymphocyte (TIL) frequencies (Martinet et al., 2011; Martinet et al., 2012; Bento et al., 2015). Similarly, while extratumoral TLS density was not a prognostic marker in pancreatic cancer patients, intratumoral TLS were an independent favorable prognosticator (Hiraoka et al., 2015). However, the relationship between tumor-associated TLS and patient outcome appears to depend on many parameters, including cancer type, disease stage, and quality of the immune infiltrate (Table 1). As discussed in detail thereafter, high frequencies of regulatory T cells in TLS can suppress antitumor immune responses (Joshi et al., 2015).

**TABLE 1:** TLS features in human cancers and effects on prognosis and/or therapy.

Tumor	TLS features	Effect on prognosis and/or therapy	References
NSCLC	Mature DC (DC-LAMP^+^)	↑ OS	(Dieu-Nosjean et al., 2008; Germain et al., 2014; Goc et al., 2014; Singhal et al., 2016)
	Th1 CD3^+^ CD4^+^	Mature DC not detected in metastasis	
	CD3^+^CD8^+^ T cells		
	B cells Ki67^+^, plasmacells		
	Neutrophils (APC-like)		
Breast cancer	nd	Association with higher tumor grade	(Figenschau et al., 2015; Sofopoulos et al., 2019; Zhang et al., 2021)
	↑ Treg	↑ risk of recurrence and death	(Gobert et al., 2009; Miao et al., 2021; Song et al., 2019)
	↑ B cells, ↓ Treg	↑ OS	(Germain et al., 2021)
Ovarian cancer (HGSC)	Plasmacells	nd	(Kroeger et al., 2016)
	CD8^+^ T cells		
	Neutrophils	nd	(Montfort et al., 2017)
Prostate cancer	↑ Th1, CD8^+^	Association with spontaneous remission	(Garcia-Hernandez et al., 2017)
	↓ Treg		
Colon cancer	Tfh	nd	(Schweiger et al., 2016)
	FDC and GC B cells	↓ risk of recurrence with mature TLS	(Posch et al., 2018)
	↑ Th2 and macrophages	↑ risk of recurrence	(Yamaguchi et al., 2020)
	↑ Tfr (Bcl6^+^)		
Pancreatic cancer	Intratumoral localization with Th1-Th17 signature	↑ OS and DFS	(Hiraoka et al., 2015)
Gastric cancer	↑ eTreg PD-1hi	Disease progression upon nivolumab tx	(Kamada et al., 2019)
HCC	↑ T CD8^+^, B cells	↓ risk of early tumor recurrence	(Li et al., 2020)
	↓ Treg		

NSCLC, non-small cell lung cancer; OS, overall survival; DFS, disease free survival; HCC, hepatocellular carcinoma; nd, not determined; HGSC, high grade serous cancer; APC, antigen presenting cell; eTreg, effector Treg.

For this reason, we believe that greater knowledge about TLS development, composition, and function may offer new therapeutic opportunities to modulate antitumor immunity. In recent years the tumor microenvironment has been considered as an active niche in a state of dynamic evolution (Rosenthal et al., 2019). In fact the complex cross-talk between the neoplastic sub-clones and immune cells represent a crucial event for neoplastic progression (Locy et al., 2018). Although tumor-associated TLS are usually associated with higher densities of CD8^+^ intratumoral lymphocytes, multivariate studies performed on NSCLC and colorectal cancer series have demonstrated that their prognostic value is independent of TIL density (Di Caro et al., 2014; Goc et al., 2014). Furthermore, intratumoral TLS are more significantly associated with longer patient survival than peritumoral ones in pancreatic adenocarcinoma (Hiraoka et al., 2015) and also in early stage hepatocarcinoma (Li et al., 2020). Wirsing et al. reported that oral squamous cell carcinoma patients with high TLS density tended to survive longer, although this was not statistically significant (Wirsing et al., 2014).

Bertucci et al. detected a higher expression of TLS signatures with higher sensitivity to immune checkpoint inhibitors in inflammatory breast cancer (Bertucci et al., 2021). In breast cancers, TLS were not significantly correlated with clinical variables (patient age, tumor size) but were considerably influenced by other pathological parameters (e.g., tumor differentiation grade, lymphovascular invasion status and pTNM stage), confirming their key role in the neoplastic progression (Zhang et al., 2021). Patients with HER-2^+^ breast carcinoma featuring TLS, that were treated with chemotherapy and/or HER-2 targeted therapy, showed a good treatment response to be attributed also to active antitumor immunity of TLS (Luen et al., 2017).

Some studies highlighted a correlation between TLS and a higher frequency of lymph nodal metastasis in high grade breast carcinoma, suggesting their unfavorable prognostic value and, at the same time, the necessity of associating TLS with other prognostic parameters (Figenschau et al., 2015; Sofopoulos et al., 2019).

The prognostic value of TLS has been particularly well-explored in colorectal cancer (Schweiger et al., 2016; Posch et al., 2018; Trajkovski et al., 2018). Yamaguchi et al. performed experiments aimed to investigate the cellular composition of TLS and to assess which cytotype correlates with disease recurrence. Five types of TLS (GC-rich, B-cell rich, follicular DC rich, Th (CD4) rich, and CTL (B/Th rich) were identified in term of cellular composition, and the density of CD4^+^ T cells and macrophages was associated with a higher rate of relapse in patients (Yamaguchi et al., 2020). On the contrary, Posch et al. delineated only three types of TLS, based on the composition of the aggregates and the functional state of a single cytotype within TLS, distinguishing the early TLS (dense aggregates with undifferentiated FDC); Primary follicle-like TLS (cluster of B cells with FDC networks but without GC); and follicle-like secondary TLS (with GC), and showing that a high percentage of mature TLS correlated with a better prognosis (Posch et al., 2018). In triple negative breast cancer, Seow et al., through a multiplex imaging analysis, identified a high density of plasma cells within TLS, suggesting a functional role for B cells in outcome (Seow et al., 2020).

Schlosser et al. demonstrated that, in esophagogastric adenocarcinoma patients, B cells within TLS produced tumor-specific antibodies, confirming their functional role in decelerating tumor growth (Schlosser et al., 2019).

## Cell Mediated and Antibody Responses in Cancer: Connection With Tertiary Lymphoid Structures

The role of adaptive immune responses in controlling tumor onset and evolution is now well established and supported by substantial preclinical and clinical evidence. Cells from nascent tumors can indeed be recognized by cytotoxic T cells since they display on their membrane peptide epitopes associated with major histocompatibility complex-class I (MHC-I) molecules that can drive tumor rejection. The intrinsic genetic instability of transformed cells is a major source of tumor antigens and, as such, the trigger of tumor specific adaptive immune responses. Nevertheless, such responses can promote the selection of poorly immunogenic tumor cell variants that escape immune control and can ultimately lead to cancer development (Mittal et al., 2014; Schumacher and Schreiber, 2015).

Animal models have highlighted the protective role of T cells and IFN-γ in tumor development. Mice lacking the adaptive arm of the immune system or unresponsive to IFN-γ (IFNGR1 and STAT1 ko) show increased susceptibility to both carcinogen-induced and spontaneous tumorigenesis (Kaplan et al., 1998; Smyth et al., 2000; Shankaran et al., 2001). In human cancers the infiltration of both CD8^+^ and CD4^+^ T lymphocytes is a common characteristic of tumors that have a favorable prognosis (Fridman et al., 2017; Paijens et al., 2021). CD8^+^ T cells exert their antitumor function both directly, by killing tumor cells expressing tumor-associated antigens on MHC-I molecules, and indirectly, by releasing proinflammatory cytokines (such as IFN-γ and TNF-α) that activate other leukocytes and sustain their antitumor activities. The infiltration of CD8^+^ T cells with an effector memory phenotype (TEM) positively correlates with overall and disease free survival in both colorectal and breast cancer patients (Pages et al., 2005; Galon et al., 2006; Bindea et al., 2013; Ahmadvand et al., 2019). More recently, a protective role of tissue resident memory (TRM) CD8^+^ T cells, that do not recirculate in blood, has emerged in several solid cancers. In both human and murine tumors, infiltration of CD103^+^ CD8^+^ T cells (a marker for TRM) is associated with improved survival (Paijens et al., 2021). Interestingly, non-tumor-specific TRM have been identified in tumor masses that can contribute to the antitumor immune response via a bystander effect (Simoni et al., 2018; Rosato et al., 2019). In mouse models of melanoma and colon carcinoma, boosting of virus-specific TRM via peptide vaccination resulted in recruitment and activation of both natural killer and dendritic cells and in upregulation of programmed death-ligand 1 (PD-L1), thus sensitizing tumors to immune checkpoint inhibitors-based immunotherapy (Rosato et al., 2019).

CD4^+^ T cells represent a heterogeneous subset of lymphocytes with high phenotypical and functional plasticity and can be considered as the playmakers of the immune system. Several T helper cell subsets have been characterized, each one featuring a specific molecular signature, that are required for both priming of CD8^+^ and B lymphocytes and for optimal antigen presentation by dendritic cells through the CD40L-CD40 axis. In line with their heterogeneity, CD4^+^ T cells can play important yet opposing roles in antitumor immunity. T helper 1 (Th1) polarized CD4^+^ T cells sustain inflammation and cytotoxic cell function and survival through production of IFN-γ and other cytokines (IL-15, IL-12) and are usually associated with a good prognosis in several cancer types (Bindea et al., 2013; Chraa et al., 2019). Likewise, detection of Tfh has been associated with improved prognosis in breast and colorectal cancers (Paijens et al., 2021). Tfh presence is indeed suggestive of TLS, and thus of antitumoral cytotoxic and antibody responses. On the other hand, Foxp3-expressing Tregs are the CD4^+^ T cell subset devoted to maintaining immune homeostasis by suppressing the effector functions of both innate and adaptive immunity. Tregs are expanded in tumors where they promote disease progression by inhibiting antitumor immunity through several mechanisms, including release of immune-suppressive cytokines (IL-10, TGF-β) and deprivation of nutrients, growth factors (IL-2) and costimulatory signals. Treg infiltration is predictive of poor prognosis and their targeted depletion is regarded as an appealing strategy for immunotherapy, even though clinical attempts have not achieved substantial efficacy so far. On the one side, the Treg-depleting activity of anti-CTLA4 antibodies described in mouse models (Selby et al., 2013; Simpson et al., 2013) has not been replicated in human cancers (Sharma et al., 2019) and subsequent attempts of targeting tumor infiltrating Tregs by an anti-CCR4 antibody were limited by concomitant depletion of effector CD4^+^ and CD8^+^ T cells (Kurose et al., 2015). Recently, Campbell and coworkers proposed a CCR8-dependent depletion of tumor infiltrating Tregs based on the highly restricted expression of this receptor on tumor infiltrating Tregs as compared to both their circulating counterpart and to intratumoral effector T cells in different human tumors. Treatment of tumor bearing mice with the anti-CCR8 antibody was highly specific to tumor infiltrating Tregs, sparing both intratumoral effector T cells and Tregs in non-tumor tissues and ultimately enabling effective and long-lasting antitumor immunity (Campbell et al., 2021).

A strong endorsement to the significance of functional T cell responses in cancer control came from the licensing of immune checkpoint inhibitors (ICIs) for immunotherapy. ICIs are monoclonal antibodies targeting molecules involved in downregulation of effector T cell functions, typically CTLA-4, PD-1 and its ligand PD-L1 (Pardoll, 2012; Topalian et al., 2015). CTLA-4 and PD-1 expression is upregulated upon T cell activation and the latter has been associated with T cell functional exhaustion in both chronic viral infections and cancer (Barber et al., 2006; Keir et al., 2008). Hence, preventing PD-1 interactions with its ligands PD-L1 and PD-L2 in the tumor microenvironment is expected to restore the cytotoxic functions of exhausted tumor-specific T cells and has achieved significant success in the management of advanced stage solid tumors.

Despite having been disregarded by the majority of studies on prognosis and response to immunotherapy so far, accumulating evidence indicates that B lymphocytes can exert both anti- and pro-tumoral activities. Similarly to T cells, B cells are indeed highly versatile leukocytes endowed with both effector and modulatory functions. Besides being the source of antibodies, B cells can effectively prime CD4^+^ T cells as professional APC (Bruno et al., 2017; Hong et al., 2018; Hua and Hou, 2020) and participate in tolerance maintenance as IL-10 producing B regulatory cells (Bregs) (Rosser and Mauri, 2015). Infiltration of B cells is often associated with a good prognosis in different tumor types and can be predictive of response to immunotherapy. Both in melanoma and soft tissue sarcoma patients, B cell abundance and the concomitant detection of TLS was associated with improved survival and to a better response rate to ICI therapy with the anti-PD-1 pembrolizumab (Helmink et al., 2020; Petitprez et al., 2020). Of note, in the sarcoma study, B cells appeared to be the strongest prognostic factor, irrespective of high or low CD8^+^ T cells and other cytotoxic cell content (Petitprez et al., 2020). Evidence of antibody response induction has been identified in different tumor types, including melanoma and breast cancer (Garaud et al., 2019; N et al., 2021). Insights on the significance of antibody responses in cancer came from studies on breast cancer after the approval of the therapeutic antibody trastuzumab. Trastuzumab targets the HER2 receptor, overexpressed by a subtype of breast tumors, and has been shown over the years to induce both antibody-dependent cell cytotoxicity (ADCC) and antibody-dependent cell phagocytosis (ADCP) by engaging the Fcγ receptors on natural killer cells, monocytes and macrophages (Park et al., 2010; Tsao et al., 2019; Ehlers et al., 2021; Upton et al., 2021). The detection of high levels of HER2-specific autoantibodies in a cohort of breast cancer patients positively correlated with the infiltration of B cells (CD20^+^) and of CXCL13^+^ cells, suggestive of TLS presence (Sato et al., 2021).

The induction and persistence of functional tumor specific adaptive immune responses can be a major barrier to disease progression. It is clear from the above discussion that both T and B lymphocytes are activated in the presence of cancer cells and both arms of the adaptive immune system play a role in disease progression and response to therapy. Several retrospective studies in humans have associated the presence of TLS with good prognosis and improved responses to ICI-based immunotherapy in distinct cancer types (Fridman et al., 2017; Sautes-Fridman et al., 2019). The beneficial effect of TLS seems mostly related to their qualitative cellular composition, with Th1-polarized CD4^+^, effector memory CD8^+^ T cells and mature dendritic cells associated to the best outcomes in colorectal and lung cancers (Dieu-Nosjean et al., 2008; Di Caro et al., 2014; Goc et al., 2014). Whether TLS are primarily involved in induction of tumor-specific immune responses and to what extent that relates to their prognostic value has not been completely elucidated yet. The concomitant detection of dendritic cells and T and B lymphocytes in proximal vicinity may be indicative of actual antigen-specific priming occurring in TLS. In lung cancer patients, high densities of mature dendritic cells (Lamp^+^) positively correlated with infiltration of activated CD8^+^ T cells (CD69^+^ CD38^+^) and with an effector memory phenotype that localized in the tumor nest, close to target tumor cells. Notably, the concomitant detection of dendritic cells and CD8^+^ T cells identified the group with a lower risk of death as compared to CD8^+^ T cell infiltration alone (Goc et al., 2014). Additional studies from the same group also reported infiltration of mature DC and B cells staining positive for the proliferation marker Ki67 and the activation induced deaminase (AID), indicating ongoing germinal center reactions. Indeed, CD138^+^ plasma cells were also detectable that specifically recognized tumor antigens in half of patients (Germain et al., 2014). As for CD8^+^ T cells, the best clinical outcome in terms of survival was observed in patients showing concomitant infiltration of B cells and dendritic cells both in early and late stage tumors (Germain et al., 2014). In high grade ovarian cancer patients, TLS with high density of plasma cells actively synthesizing IgG were described. Again, plasma cell infiltration correlated with cytotoxic T cells infiltration and dictated their prognostic benefit (Kroeger et al., 2016).

**FIGURE 1:**
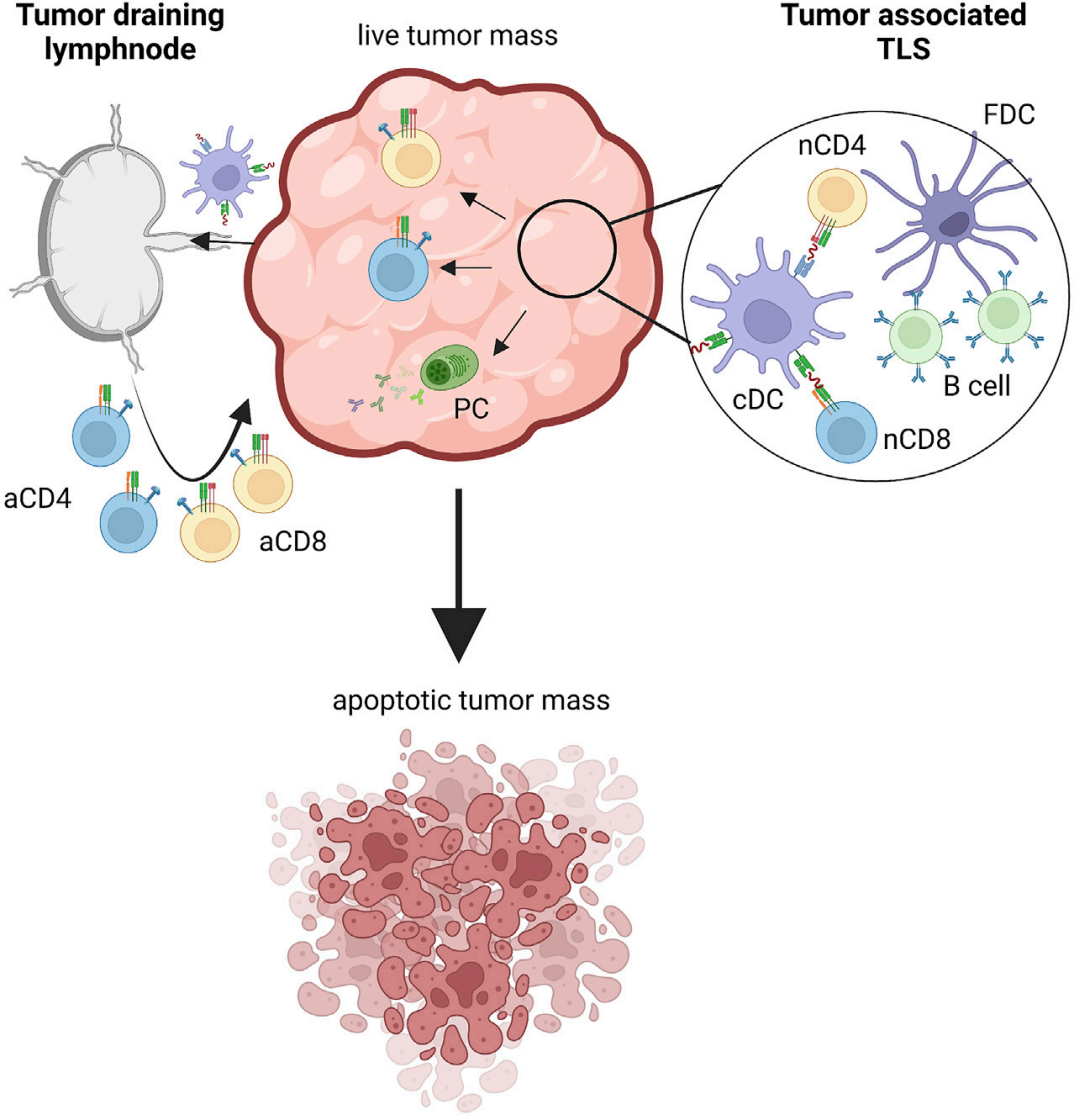
Inductive sites of tumor-specific immune responses. Tumor cells express tumor associated antigens (TAAs) sampled by dendritic cells that migrate to tumor draining lymph nodes where adaptive immune responses are triggered. Tumors also feature tertiary lymphoid structures (TLS) where tumor specific T and B cells can be primed. In both cases, activated tumor-specific CD4^+^ and CD8^+^ T cells migrate within the tumor mass and attack transformed cells, ultimately inducing apoptotic cell death. Also plasma cells participate in antitumor immunity by producing tumor specific antibodies. aCD4, activated CD4 T cell; aCD8, activated CD8 T cell; nCD4, naive CD4 T cell; nCD8, naive CD8 T cell; PC, plasmacell; cDC, conventional dendritic cell; FDC, follicular dendritic cell. Created with BioRender.com.

These studies suggest that specific and complete immune responses can be induced at the tumor site and that TLS facilitate interactions between innate and adaptive immune cells (Figure 1). These observations are supported by some studies that implied the occurrence of active immune responses in ectopic lymphoid structures found in autoimmune conditions and infections. For instance, splenectomized mice lacking SLO and Peyer’s Patches (Lymphotoxin *α* −/−) can mount functional antigen-specific CD8^+^ T cell and antibody responses upon influenza virus infection that occur at the inducible bronchus associated lymphoid tissue (iBALT) and sustain viral clearance (Moyron-Quiroz et al., 2004). Furthermore, iBALT-primed T and B cells develop a memory phenotype and provide protection against viral challenge (Moyron-Quiroz et al., 2006). Antigen-specific priming in the absence of canonical SLO has also been reported in tumor models. Schrama and colleagues grew in splenectomized LTα^−/−^ mice B16 derived tumors that can be specifically targeted with LTα, a well-known lymphoid tissue inducer. The authors documented delayed growth of subcutaneous tumors and complete protection from pulmonary metastasis. Importantly, CD8^+^ T cells specific for the tumor associated antigen TRP-2 were detectable by *in situ* tetramer staining only within tumors where TLS had been induced (Schrama et al., 2008). Similarly, antitumoral activity was shown for adoptively transferred naive T cells even when lymphocyte egress from LN was blocked by FTY720 (Thompson et al., 2010; Peske et al., 2015). These results suggest that antitumor immune responses likely induced in TLS can restrain tumor growth and control disease evolution by protecting from metastatic spread. More recently, the generation of TLS has been associated with the composition of the gut microbiome, whose involvement in tumor development and immunity has raised much interest. Overacre-Delgoffe and coworkers assessed the occurrence of TLS in a carcinogen induced colorectal cancer model in the presence of the bacterial species *Helicobacter hepaticum* (Hhep). Hhep colonized mice developed smaller tumor numbers as compared to controls, survived longer and developed intratumoral TLS dominated by Tfh responses that appear to be the correlate of protection. Indeed, both TLS formation and the effect on disease progression were abrogated in mice specifically devoid of Tfh and rescued upon Hhep-specific CD4^+^ T cell adoptive transfer (Overacre-Delgoffe et al., 2021).

## Stromal Cell Composition and Architecture in Tertiary Lymphoid Structures Formation

Within the tumor microenvironment the local cross-talk between immune cells and stromal elements leads to the production of a series of pro-inflammatory cytokines and TNF receptor family components that determines the formation of TLS (Le Hir et al., 1996; Matsumoto et al., 1996; Endres et al., 1999; Forster et al., 1999; Lorenz et al., 2003; Cupedo and Mebius, 2005). Different non-hematopoietic stromal cells, namely fibroblasts, blood and lymphatic endothelial cells, pericytes, and epithelial cells, participate variably to TLS development (Buckley et al., 2015). Several studies have demonstrated a key role of the stromal elements, in particular fibroblasts, in chronic inflammation by the activation of pro-survival and retention signals. In fact, fibroblasts showed to promote the local recruitment of immune cells at the inflammatory site and support the maintenance of the inflammatory state by producing various factors, such as B-cell survival factors (BAFF) and inflammatory chemokines (e.g., IL-8, CCL5, CXCL1). However, fibroblasts can also participate in the formation of TLS through their capability to produce other chemokines, such as CXCL12, CCL21 and CXCL13 (Filer et al., 2008; Flavell et al., 2008; Barone et al., 2012). Soluble molecules produced by stromal cells have been harnessed to drive TLS formation for therapeutic purposes (Johansson-Percival and Ganss, 2021). For instance, in a neuroendocrine tumor mouse model, targeting endothelial cells and activated tumor infiltrating macrophages (CD68^+^) with the LN-inducing cytokine LIGHT (TNFSF14) induces production of CCL21, TNFα, and IL1β that, overall, drive T/B cell recruitment and formation of mature TLS (Johansson-Percival et al., 2015; Johansson-Percival et al., 2017). In a distinct approach, genetically engineered DC overexpressing Tbet, and thus producing high levels of IFNγ, TNFα and IL36γ, have been shown to induce TLS in murine colon cancer even in the absence of peripheral LN (Weinstein et al., 2017).

In 2004 Cupedo et al. highlighted the crucial role of stromal cells in the development of TLS by performing intradermal injections of cell suspensions obtained from neonatal lymph nodes, which seemed to promote the formation of ectopic lymphocyte aggregates resembling lymph node-like follicular structures. Interestingly, the major cellular actors strictly involved in this process were the mesenchymal elements showing the capability to engage and retain host-derived T and B lymphocytes organized into two distinct compartments (Cupedo et al., 2004). Subsequently, Suematsu et al. used a mouse model and performed the implantation of stromal cell scaffolds under the kidney capsule, demonstrating the generation of lymphoid structures. These structures, described as artificial lymph nodes, recapitulated the morphological characteristics of lymph node parenchyma and proved to be able to support immune responses (Suematsu and Watanabe, 2004). Furthermore, other studies supported the pivotal contribution of stromal cells in the development of ectopic lymphoid structures during chronic inflammation. In a model of atherosclerosis, the investigators described the formation of adventitial aortic tertiary lymphoid organs, due to the activation of mouse aorta smooth muscle cells expressing both VCAM-1 and chemokines CXCL13 and CCL21 (Grabner et al., 2009).

**FIGURE 2:**
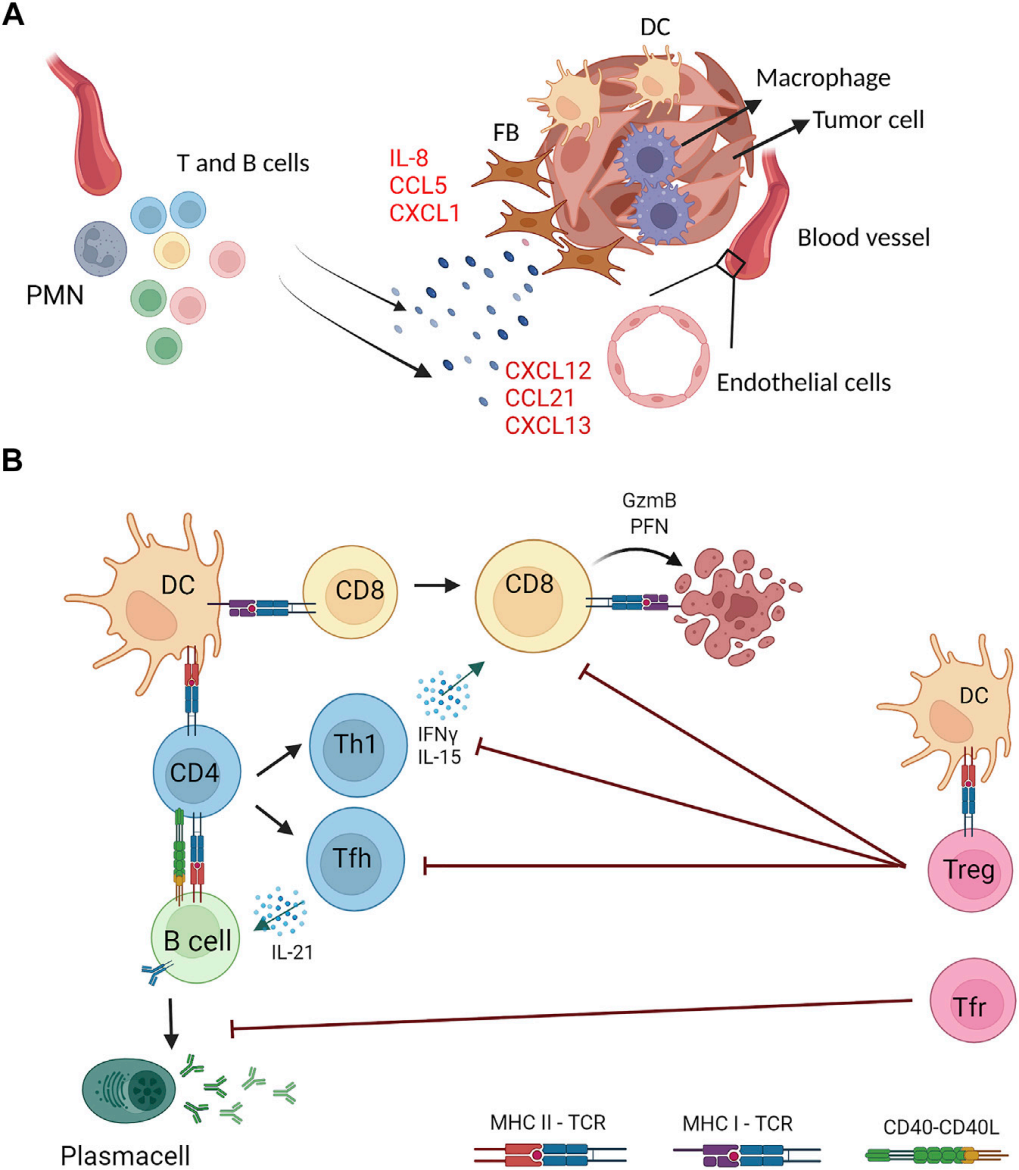
Stromal and immune cells crosstalk in the generation and functions of tumor associated TLS. **(A)** Stromal components of tumor masses (fibroblasts, endothelial and myeloid cells) are the source of proinflammatory cytokines (e.g., IL-8) and chemokines (e.g., CXCL13) that drive recruitment and activation of lymphocytes and other myeloid cells (e.g., neutrophils) at the tumor site that generate organized lymphoid structures **(B)** Tumor associated TLS feature multiple interactions between lymphoid and myeloid cells for induction of tumor specific immune responses. Dendritic cells (DC) and B cells can present TAA on MHC class I and class II molecules to CD8^+^ and CD4^+^ T cells expressing the cognate T cell receptor (TCR) and, concomitantly, provide costimulatory signals by CD40^−^CD40L axis. Upon antigen recognition, CD8^+^ T cells differentiate into cytotoxic cells that kill tumor targets by perforin (PFN) and Granzyme B (GzmB). CD4^+^ T cells can differentiate into several T helper subsets, including T helper 1 (Th1) and T follicular helper (Tfh) that sustain, respectively, cytotoxic and B cell responses through the production of specific cytokines (IFNγ, IL-15, IL-21). Activated B cells differentiate into antibody secreting plasma cells. TAA-specific CD4^+^ T cells include regulatory T cells that, once activated, counteract effector CD4^+^ and CD8^+^ T cells. A specialized subset of Treg with a follicular phenotype (Tfr) inhibit B cell responses. Created with BioRender.com.

Overall, as a consequence of persistent antigen presentation due to chronic inflammatory stimuli, stromal cells acquire lymph node-like properties that allow them to recruit, activate and prime adaptive immune cells (Figure 2A). A variety of inflammatory stimuli, either infectious, autoimmune or tumor-related, have proved to be responsible for the formation of such antigen-specific lymphoid aggregates. Thus, in the setting of a solid neoplasm, TLS formation accounts for the promotion of a tumor-specific immune response that elicits a targeted intratumoral adaptive immunity. Knowledge of these processes helps define TLS as promising prognostic biomarkers to stratify the patient’s overall survival rate and may soon contribute to the development of novel immunotherapeutic agents for the treatment of different solid neoplasms (Colbeck et al., 2017a).

Even during development, TLS stroma presents a large degree of plasticity (Barone et al., 2016). Early anlagen mesenchyme can differentiate upon specific stimuli into diverse and highly specialized stroma, which then creates functional micro domains within the TLS (Asam et al., 2021). TLS non-vascular stroma is largely represented by fibroblastic reticular cells (FRC), a fine network of canaliculi forming fibroblasts that display unique functional properties, including the ability to support the dramatic anatomical remodelling required to adapt TLS to the lymphocyte influx occurring in response to antigen stimulation (Perez-Shibayama et al., 2019). The mechanism underpinning the physical plasticity of the TLS fibroblasts has been identified in the SLO in the interaction between podoplanin, a glycoprotein broadly expressed on FRC, and its receptor CLEC2 expressed by DC, first incomers in the TLS upon antigen exposure, during inflammation or upon immunization (Acton et al., 2014). The adaptability of the stroma during the immune response has been deemed critical to enable expansion of the B follicle required to accommodate the GC reaction upon immunization. Plasticity of the fibroblasts in TLS can therefore be defined as the most critical property required to shape an efficient immune response (Barone et al., 2016; Asam et al., 2021).

## Myeloid Cells in Tertiary Lymphoid Structure Formation

TLS are organized formation of immune cells, which arise in tissue during chronic inflammation (e.g., autoimmunity, allograft rejection, chronic inflammation, and cancer) (Manzo et al., 2010; Thaunat et al., 2010; Neyt et al., 2012; Colbeck et al., 2017a). Similarly to lymph node and SLO, TLS displayed an inner area of CD20^+^ B cells surrounded by CD3^+^ T cells (Sautes-Fridman et al., 2019). Even though TLS are populated by a variety of T cells (CD4^+^ follicular helper T cells, CD8^+^ cytotoxic T cells, CD4^+^ T helper 1 and regulatory T cells) (Gu-Trantien et al., 2013; Goc et al., 2014; Hennequin et al., 2016; Kroeger et al., 2016) and B cells, TLS are also populated by distinct types of myeloid cells. In particular, follicular dendritic cells (FDC), characterized by the expression of CD21, localize in the inner B zone (Bergomas et al., 2011) and dendritic cell-lysosomal associated membrane protein (DC-LAMP^+^, also known as CD83^+^ DC) preferentially distribute in T cell zone and activate T cell (McMullen et al., 2010). DC may also accomplish other functions in TLS organization. Indeed, it has been suggested that antigen-presenting DC, through the activation of T cells, are sufficient to TLS induction (Ludewig et al., 1998; Neyt et al., 2012). In line with this, the depletion of DC determined the loss of existing TLS, indicating the importance of DC for structural organization and maintenance of TLS, probably through the production of chemokines (e.g., CXCL13) or by the continuous activation of T cells (GeurtsvanKessel et al., 2009; Halle et al., 2009; Muniz et al., 2011). The presence of DC in TLS, as well as of other immune cell types, has been associated with better prognosis in cancer (Goc et al., 2014; Sautes-Fridman et al., 2019).

Cancer-associated TLS can also contain scattered CD68^+^ macrophages (Dieu-Nosjean et al., 2008; Schumacher and Thommen, 2022), and the function of these macrophages has not been fully characterized. One hypothesis is that they function as scavenger cells, as it is in SLO, where T cell zone macrophages (TZM) acted as the only professional scavenger cells clearing up apoptotic cells (Baratin et al., 2017). During atherosclerosis, M1-polarized macrophages act as potential lymphoid tissue inducer cells (LTi) (Guedj et al., 2014). In this context, macrophages were identified as the inducer cells that enhanced the expression of chemokines by vascular smooth cells. Interestingly, this function in M1-polarized macrophages was independent of lymphotoxin-a1b2 (LTbR) signaling (Guedj et al., 2014). The ability of macrophages to induce TLS has been shown in another pathological context. Indeed, using a model of *Salmonella* colitis, Koscso and colleagues demonstrated that only intestinal mucosa resident CXCR1^hi^ macrophages act as the APC responsible for the recruitment of CD4^+^ T cells and B cells at the site of *Salmonella* invasion, leading to the formation of TLS and to the local pathogen specific IgA response (Koscso et al., 2020).

Neutrophils can infiltrate TLS and have been reported to infiltrate the TLS found in the omental metastasis of patients with high-grade serous ovarian cancer (HGSOC) (Montfort et al., 2017), and the TLS of patients with prostate cancer (Garcia-Hernandez et al., 2017). The function of neutrophils in this context has not been characterized. Neutrophils present in secondary lymphoid organs can influence antigen presentation and B-cell antibody response (Lok and Clatworthy, 2021). Indeed, neutrophils in the spleen provide helper signals to B cells through the production of BAFF, APRIL, and IL-21 (Puga et al., 2011). In lymph nodes, neutrophils expressing high level of major histocompatibility complex II (MHCII) are mostly located in proximity of T cells and natural killer cells, suggesting a role in CD4^+^ T cell activation (Lok et al., 2019). In early-stage NSCLC patients, neutrophils that can acquire APC-like features and activate CD4^+^ T cells and CD8^+^ T cells have been identified (Singhal et al., 2016). However, the precise role of neutrophils in TLS has not been elucidated and in our opinion deserves further investigation.

## Regulatory T Cells in Tertiary Lymphoid Structures: Metabolic Aspects Underlying Their Fitness in the Tumor Microenvironment

In accordance with their well-defined and above discussed role in tumors, a detrimental role in antitumor immunity has been documented in studies focusing on TLS-associated Tregs. In mouse models of fibrosarcoma and lung adenocarcinoma, Treg depletion triggers TLS and increases T cell activation with ensuing tumor control (Hindley et al., 2012; Colbeck et al., 2017b). In human breast and prostate cancers, the presence of Foxp3^+^ cells in TLS is associated with adverse clinical outcomes (Gobert et al., 2009; Garcia-Hernandez et al., 2017; Germain et al., 2021). Interestingly, in a study on breast cancer the predictive value in terms of death and relapse was specifically associated to Tregs detected within lymphoid structures rather than to those infiltrating the tumor bed. Lymphoid tissue-associated Tregs show an activated phenotype, are in a proliferative state (Ki67^+^) and in close proximity to activated DCs and T cells. This suggests that tumor antigen specific Tregs are primed in TLS, where they can directly suppress antitumor effector T cells (Gobert et al., 2009). In support of this, Joshi and colleagues have specifically demonstrated that in a mouse model of lung cancer Tregs are preferentially detected in TLS (rather than scattered in the tumor stroma) where they maintain immune quiescence. Treg depletion by diphtheria toxin administration in a transgenic model reactivates proliferation of both CD4^+^ and CD8^+^ T cells within TLS and results in enhanced tumor destruction (Joshi et al., 2015).

While Tregs are detected in tumor associated TLS in human cancers and murine models, their contribution in the formation of these structures is still not known. Considering the favorable prognostic value played by TLS in cancer, dissecting the processes and cell types involved in their formation may have a significant therapeutic impact. Tumor infiltrating Tregs show an activated phenotype characterized by the expression of PD-1 and OX40 (Piconese et al., 2014; Kamada et al., 2019; Polesso et al., 2019), that holds potential for modulating their abundance in the tumor microenvironment with therapeutic implications. In gastric cancer patients, effector Tregs (eTreg, Foxp3^high^ CD45RA^−^) express high levels of PD-1 that impairs anti-PD-1 based therapy, inducing rapid disease progression. Kamada and colleagues observed hyperprogressive disease in 10% of patients after nivolumab treatment that was associated with the accumulation of proliferating intratumoral eTreg with enhanced suppressive potential (Kamada et al., 2019). In this view, combinatorial therapies exploiting the phenotypic heterogeneity of tumor infiltrating Tregs may be a backup strategy. In mouse models of melanoma and colon cancer, anti-PD-1 treatment induced the expansion of tumor Tregs with a follicular phenotype (Follicular regulatory T cells or Tfr, Foxp3^+^ Bcl6^+^), and Tfr depletion by pretreatment with anti-CTLA4 improved tumor control. Notably, a cohort of melanoma patients undergoing sequential anti-CTLA4 and anti-PD-1 had a survival advantage over both the corresponding monotherapies or the anti-PD-1 followed by anti-CTLA4 (Eschweiler et al., 2021).

Follicular regulatory T cells are Foxp3^+^ cells that, upon antigen recognition, co-opt the differentiation pathway of Tfh and thus express the committing transcription factor Bcl6 and the chemokine receptor CXCR5, that allows relocation to germinal centers, where they modulate antibody responses (Lu and Craft, 2021). While the role of Tfr in immunization and infection models has been characterized, their significance in tumors is mostly still unknown. Nevertheless, evidence has been accumulating pointing to a central role of Tfr in modulating antitumor immunity and immunotherapy performance (Figure 2B). Increased Tfr frequencies are detected in peripheral blood of breast cancer patients (Song et al., 2019; Miao et al., 2021) and are shown to be a source of IL-10 that suppress antibodies production and promote Breg generation (Song et al., 2019). Whether a fraction of tumor infiltrating Tregs differentiate to Tfr in tumor associated TLS is not known, but a recent work supports this hypothesis. Eschweiler et al. reported that Tfr and activated Treg (4-1BB^+^) isolated from human head and neck cancer share a transcriptomic signature characterized by upregulation of genes related to activation, costimulation and suppressive functions (*TNFRSF4*, *TNFRSF18*, *TNFRSF1B*, *ENTPD1*). Importantly, a fraction of activated Tregs shared TCR with Tfr, indicating a developmental relationship between the two subsets. A similar pattern was identified in murine melanoma derived Treg/Tfr, suggesting that Tfr may actually derive from tumor infiltrating Tregs that in TLS are activated by tumor-specific antigens (Eschweiler et al., 2021). Tfr represent an attractive therapeutic target given their interactions with immune ICIs and their increased suppressive functions over Bcl6^−^ Tregs (Sage et al., 2014a; Sage et al., 2014b; Eschweiler et al., 2021).

Since the anti-tumor immune response occurs locally within the tumor mass and is not restricted to the activation of effector T cells in draining secondary lymphoid organs, the cellular composition and the metabolic events occurring at the TME contribute to TLS formation, thus impacting on tumor suppression and therapeutic response. The B-cell mediated antigen presentation to CD4^+^ T cells occurring in the TLS is finely regulated by different external stimuli and by the TME conditions. In this context, the TLS-B cell interaction is crucial to modulate the early phases of T cell activation, their co-stimulatory properties and/or cell exhaustion, driving their lineage decision. More in detail, it has been described that TLS-B density positively correlates with a specific CD4^+^ T cell signature characterized by the hyper-expression of pro-inflammatory genes including POU2AF1, which encodes for the transcriptional coactivator OCA-B (also called OBF-1) that directly stimulates the IFN-γ and IL-2 promoter activities and is required for the *in vivo* generation of CD4^+^ memory T cells (Brunner et al., 2007; Shakya et al., 2015). Recently, Germain and colleagues observed that high TLS-B density in NSCLC subjects negatively correlates with the exhausted CD4^+^ T cell compartment, expressing several Treg-cell associated genes and molecules, including CD5, CD25, GITR, and Tim-3. Conversely, they found a higher percentage of CD4^+^Foxp3^+^ Tregs in NSCLC subjects with low TLS-B density compared with those expressing high TLS-B density. This shows that high TLS-B cell density correlates positively with naïve, central memory and effector CD4^+^ T cell number but negatively with exhausted T cells and Treg cells (Germain et al., 2021).

The metabolism of the TME represents a key point in the polarization of the tumor-specific immune response. There exists evidence that low-glucose and high-lactate environment within the TME constraints T cell effector function, while promoting Treg generation and immunosuppression (Wang et al., 2018). Treg cells in tumors rely on a combination of glycolysis and fatty acid synthesis and oxidation, which allows their survival and proliferation in the hostile tumor environment (Pacella et al., 2018). A recent study has further elucidated that high-glucose conditions impair the function and stability of Tregs. Interestingly, Tregs have evolved to benefit from the symbiosis with tumors by utilizing the glycolytic by-product lactic acid (LA); indeed, they incorporate lactate-derived carbon into phosphoenolpyruvate (PEP) to provide upstream glycolytic intermediates essential for proliferation. This offers the opportunity to decrease their need for glucose, thus preserving Foxp3 induction and suppressive function (Watson et al., 2021). In addition, very recently the link between LA and PD-1 expression in TILs has been clarified; more in detail, LA upregulated PD-1 expression by eTregs through MCT1, which is controlled by Foxp3 under a low-glucose and high-LA TME. Conversely, the effect of high LA concentration on PD-1 expression by CD8^+^ T cells shows an inverse trend. This means that the efficacy of PD-1 blockade against tumors is improved by targeting the LA metabolism of Tregs (Kumagai et al., 2022). Zappasodi and colleagues recently showed that CTLA-4 blockade promotes immune cell infiltration and metabolic fitness especially in glycolysis-low tumors. Accordingly, inhibition of tumor glycolysis ameliorates the ability of CTLA-4 blockade to induce loss of Treg stability associated with the development of anti-tumor immunity (Zappasodi et al., 2021). Moreover, increased lipid metabolism in intratumoral Tregs, which is known to boost their suppressive function, is a shared event in human and mouse cancer, occurring through the CD36 up-regulation. CD36 supports mitochondrial fitness and biogenesis *via* a peroxisome proliferator-activated receptor-β (PPAR-β)-dependent mechanism by modulating NAD^+^ levels that allow Tregs to adapt to a lactic acid-enriched TME (Wang et al., 2020). Recently, it has been reported that inhibition of fatty acid binding proteins (FABP)5, one of the lipid chaperones required to facilitate uptake and intracellular lipid trafficking, causes mitochondrial damage, mtDNA release and consequent activation of the cGAS-STING-dependent type I IFN signaling; this promotes greater suppressive capacity, a phenotype also evident in tumor Tregs (Field et al., 2020).

Recent studies have revealed the importance of Blimp1 in the regulation of effector Treg (eTreg) and Tfr cell stability and suppressive function. Tfr cells express Foxp3 and belong to eTregs, and share many features with Tfh cells, as high expression levels of PD-1 and CXCR5, which allow them to traffic to B-cell follicles in response to CXCL13 (Garg et al., 2019; Shen et al., 2019). To date, few reports reported that Tfr cells are increased in cancer patients (Cha et al., 2018; Li et al., 2019), but their mechanism of action in the tumor is still unclear. However, the formation of TLS and the increased proportion of Tfh and B cells within the tumors, as observed in mice with Blimp1-specific deletion in Treg cells, are associated with favorable outcomes and with an increased response to immunotherapy in several tumors (Dixon et al., 2021).

## Discussion

The development of specific immune responses is a common characteristic of tumor hosts, triggered by the inflammation resulting from recognition of antigenically aberrant transformed cells. As to whether tumor specific immune responses can be triggered locally has been long a matter of debate and the discovery of tumor associated TLS and of their composition represents a central point of discussion. Animal models of secondary lymphoid organs deficiency, such as the splenectomized LTα −/− mice, have shown that the presence of tumor associated TLS is sufficient to induce effective antitumor immune responses (Schrama et al., 2008). Some characteristics of tumor associated TLS, such as the presence of mature dendritic cells, T cells and actively proliferating B cells (Ki67+) in close proximity, can be indicative of ongoing antigen specific activation. Despite very few studies have specifically associated TLS presence with tumor specific immunity (Germain et al., 2014; Kroeger et al., 2016; Overacre-Delgoffe et al., 2021), the strong prognostic value of these intratumoral lymphoid structures might indeed be related to their promoting effect on antitumor responses. Also, the predictive value of response to ICI-based immunotherapy further sustains a link between TLS and tumor specific immunity. Antibodies directed against PD-1, that achieved the best clinical performance amongst ICIs, are thought to alleviate the suppressive signals impinging on tumor specific effector T cells in the tumor microenvironment and thus reactivate their cytotoxic functions. Also regulatory T cells can express high levels of PD-1 and other immune checkpoint molecules in the tumor microenvironment, and this may undermine the efficacy of ICIs as observed in gastric cancer (Kamada et al., 2019). It is therefore important to include TLS detection and characterization in tumor specimen analysis to improve the clinical management of the disease.

Regulatory T cells are found in tumors both scattered within the mass and associated with TLS. Studies on TLS-associated Tregs overall fit with their detrimental role in cancer and provide insight on their tumor promoting activities. Treg with a follicular phenotype (Foxp3^+^ Bcl6^+^ CXCR5^+^) have been the focus of recent studies, that highlighted a more powerful suppressive potential as compared to non-follicular Tregs (Sage et al., 2014a; Sage et al., 2014b; Eschweiler et al., 2021). In this view, follicular Tregs hold therapeutic potential to improve current therapies. There is indeed evidence that anti-CTLA-4 blocking antibodies can deplete Tfr, because of their increased expression of CTLA-4, and this improves the efficacy of subsequent administration of anti-PD-1 (Eschweiler et al., 2021). It has also been shown that tumor infiltrating Tfr share TCRs with activated Tregs and thus they derive from TAA-specific Tregs that migrate into tumors and get activated in TLS.

Tumor associated tertiary lymphoid structures represent an incredibly informative tool in tumor management and their characterization should be included in routine tumor pre- and postoperative analysis in order to provide the most accurate grade of disease management.

## Abbreviations

APC, antigen presenting cells; APRIL, proliferation-inducing ligand; BAFF, B-cell activating factor; CTLA-4, Cytotoxic T-Lymphocyte Antigen-4; DC, dendritic cells; FRC, fibroblastic reticular cell; GC, germinal center; GITR, Glucocorticoid-induced tumour necrosis factor receptor-related protein; IFNγ, interferon γ; LN, lymph node; NSCLC, non-small cell lung cancer; PD-1, Programmed Death-1; PD-L1, Programmed-Death Ligand 1; SLO, Secondary lymphoid organs; TEM, Teffector memory; Tfh, T follicular helper; Th1, T helper 1; TLS, tertiary lymphoid structures; TNFα, tumor necrosis factor α; TRM, Tissue resident memory; TME, tumor microenvironment.
